# The role of modeling in evaluation of maternal and child health programs: using the lives saved tool to help answer core evaluation questions

**DOI:** 10.1080/16549716.2021.2006421

**Published:** 2022-09-13

**Authors:** Neff Walker, Yvonne Tam

**Affiliations:** Department of International Health, Bloomberg School of Public Health, Johns Hopkins University, Baltimore, MD, USA

**Keywords:** Program evaluation, impact evaluation, maternal health, child health, newborn health, modeling

## Abstract

This paper explains how The Lives Saved Tool (LiST), a computer-based model that estimates the impact of scaling up interventions on stillbirths, maternal, neonatal and child health, can contribute to evaluations of programs being delivered at scale to improve maternal and child health. LiST can be used to estimate the impact of a program in advance, allowing planners to refine, streamline and set appropriate program targets. LiST can also be used to estimate the impact of a program, which is particularly useful given the high costs of measuring changes in population health. Finally, LiST can be used to estimate the relative contributions of different interventions or sets of interventions within programs that are found to have a positive impact. The latest version of LiST allows users to manipulate both utilization and quality of service to generate estimates of effective coverage. In addition, a new, web-based version of LiST is now available, with a simpler and more streamlined interface designed to increase accessibility to beginning users. LiST modeling can help program planners, evaluators and funders respond to core evaluation questions related to program design and impact, providing evidence to support decisions about how best to use available resources to save the lives of women and children.

## Background

There have been dramatic reductions in deaths among women and children since 2000 [1–3]. Despite this progress, there remain many deaths that could be prevented through known and effective interventions. Comprehensive evaluations of programs targeting reproductive, maternal, newborn and child health and nutrition (RMNCH&N) produce essential information needed for the continuous improvement of programs, and yet are rarely conducted [4]. The ‘Real Accountability: Data Analysis for Results’ (RADAR) project aims to improve the evidence base for program and policy decisions in RMNCH&N by providing clear guidance for designing and implementing effectiveness evaluations of large-scale programs in low- and middle-income countries (LMICs) [5]. The RADAR framework for program evaluation focuses on a set of five methodological steps, or ‘core evaluation questions’, that should guide such evaluations (Panel 1).

**Box 1.** Summary messages.

Modeling can be an important resource for those planning, evaluating and funding reproductive, maternal and newborn health and nutrition programs.
The Lives Saved Tool (LiST) brings together the best-available evidence on intervention effectiveness to estimate the numbers of maternal and child deaths that can be averted through national and subnational programs.
A web-based version of LiST (LiST Online) is now available, including specific tools for exploring available data for specific geographic areas and time periods, identifying the expected impact from selected interventions to avoid missed opportunities, and projecting the numbers of lives saved from user-defined program strategies.
LiST Online has a new user interface that builds on 15 years of applications. LiST online was designed to maximizes flexibility, access, and adaptation for use at all levels of expertise and with an expanded range of computer operating systems.
LiST can strengthen program evaluations while reducing costs of the evaluation.

**Panel 1.** The RADAR framework: five core questions for program evaluations.

Q1. Does the program focus on interventions that will have the greatest impact in the program context? Q2. Is the pathway to MNCH impact clearly developed and evidence-based? Q3. Is the program being implemented as planned, and at sufficient strength and quality to achieve expected impact? Are services being utilized? Q4. Do women and children who need interventions actually receive them? Q5. Is the expected impact of the program occurring? Why or why not?

Modeling is an essential resource for program evaluation [5,6]. This is especially true in RMNCH&N programs, which address multiple age groups and many interrelated and even inter-generational health and nutrition outcomes. Harnessing computational power has become necessary as the evidence base expands on the complex interactions among interventions and their direct and indirect effects on RMNCH&N outcomes.

Why, then, do so few program evaluations include modeling in their methodological approach?

First, most programs aim to deliver multiple interventions that address more than one disease or cause of death, while most models have historically been developed to address a single cause or disease, limiting their usefulness. For example, a model that correctly captures the impact of interventions and other external factors on pneumonia – a major cause of child mortality in most LMICs – is helpful only in planning and evaluating a pneumonia-specific program. Modeling results in this case have little relevance to broader programs (e.g. community case management programs targeting childhood pneumonia, malaria and diarrhea), and may miss even some important effects of a pneumonia program, such reductions in tetanus, diphtheria and pertussis that result from use of pentavalent vaccines (which contains Hib-vaccine) to reduce pneumonia.

Second, many models do not capture the effects of extra-program factors and interventions that determine changes in health outcomes. For example, although disease-specific models targeting child deaths due to diarrhea are likely to capture the mortality impact of achieving increases in coverage of oral rehydration solution for children with diarrhea. However, many models do not traditionally taken into account concurrent changes in coverage for other interventions (e.g. interventions to improve water and sanitation) or risk factors (e.g. stunting) that are also known to increase the risk diarrhea mortality. Although all models, by definition, are a limited reproduction of the real world, RMNCH&N models must be broad enough to capture the full range of effective interventions, major causes of deaths and risk factors and the web of interactions among them.

A third factor limiting the use of modeling in RMNCH&N program evaluations is that many models are difficult to access and use, often requiring skills beyond those of most program planners or evaluators. The models, while perhaps very accurate, have not been developed for use by most non-modelers and may require information that is not readily available at the country or program level.

In this paper, we describe the historical evolution of the Lives Saved Tool (LiST) model, and the extent to which it addresses these three constraints to the successful use of modeling in RMNCH&N program evaluations. We use the RADAR core evaluation questions (Panel 1) as a starting point. Although LiST can help program planners and evaluations answer all five questions, we focus here on two: Question 1 – ‘Does the program focus on interventions that will have the greatest impact in the program context?’; and Question 5 – ‘Is the expected impact of the program occurring? Why or why not?’.

## Overview of the lives saved tool (LiST) and the new LiST on-line

The Lives Saved Tool, or ‘LiST’, is a mathematical modeling tool that allows users to estimate the impact of coverage change on stillbirths and maternal, neonatal and child mortality in LMICs. In 2003, a group of scientists working in child survival created a spreadsheet-based model designed to answer the question, ‘Can we reach the 2015 Millennium Development Goal targets for reductions in child mortality by achieving high levels of coverage for existing interventions feasible for delivery in low-income countries?’ [7] This precursor to LiST included 23 interventions with evidence of both efficacy and feasibility for scale-up in low-resource community settings. The authors estimated that achieving high coverage with this set of interventions in the 42 countries that accounted for 90% of child deaths in 2000 could reduce child mortality by over 60%, sufficient to achieve the MDG for child survival.

This initial model introduced an overall framework for child survival that linked levels of intervention coverage with reductions in cause-specific child mortality. The authors proposed that the effect of more distal factors like education and income – while important – would be captured through changes in intervention coverage. They also took into account that both mortality levels and cause-of-death distributions would change over time as the result of changes in coverage [7].

In the intervening 17 years, the original model has been expanded, refined, and renamed as LiST. LiST has evolved from a simple spreadsheet model to a multi-faceted application operating within the Spectrum software package. Spectrum links LiST to a full demographic package with population projections for all LMICs, as well as modules used to estimate the impact of family planning and HIV/AIDS programming. [8] Panel 2 provides a brief introduction to the process of running LiST.

**Panel 2.** Running a LiST model.

Your first step in running a LiST model is to define the geographic scope for the model (country or subnational area with adequate data) and specify the year to be used as baseline. You will then develop a profile of the chosen setting in the chosen year, either by accepting default information drawn from the LiST database or by entering your own estimates. The profile includes levels of cause-specific mortality and relevant exposures, deficiencies and risk factors. It also includes coverage in the baseline year for the interventions with proven efficacy that are included in LiST (currently about 70). LiST will add to the profile by drawing on links to other databases, including those providing latest available demographic estimates. Once the baseline profile is complete, you can develop projections of the number of lives that would be saved in subsequent years by increasing coverage for one or more of the interventions. For example, to estimate the impact of scaling up diagnosis and treatment of diarrhea, pneumonia and malaria by community health workers, you would select those three interventions and indicate the target levels of coverage you expect to achieve for a given time period. LiST then uses the baseline profile as a starting point, and estimates the incremental changes in the population at risk as each intervention is scaled up, producing an estimate of the cumulative lives saved by the target year. LiST can also attribute changes in levels of risk factors and mortality to specific interventions, based on relative changes in intervention coverage, efficacy of the interventions and baseline levels of mortality and disease burden.

The health outcomes included in LiST have expanded over time from overall to cause-specific child mortality, maternal mortality, stillbirths, birth outcomes (low birth weight, small for gestational age, prematurity), maternal anemia, and stunting and wasting. This has meant the addition of many additional interventions, all supported by evidence of efficacy, and refinements in the inter-relationships between family planning programming and birth-related risks for mothers and children. A full description of the model, as well as a visualizer showing the current LiST structure and assumptions underlying links between interventions, risk factors and health outcomes can be seen at https://www.livessavedtool.org/.

LiST data and assumptions have expanded over time to reflect the evolving RMNCH&N evidence base. The original list of 23 child survival interventions has expanded to include interventions across the full RMNCH&N continuum of care. The underlying framework now includes not only interventions that have a direct effect on mortality, but also those that affect mortality indirectly by changing levels of risk factors. For example, LiST now includes the complex relationships between nutrition and the health of women and children, including measures and interventions related to growth [9,10]. In addition, LiST now provides the option of using quality-adjusted estimates of coverage for antenatal and childbirth interventions that take into account measures of service utilization and readiness to deliver care [11,12].

A key feature of LiST is that the model was developed so that it reflects the interactions of over 80 different interventions that have been proven efficacious in improving the health of women and children. This includes efficacy in reducing cause-specific mortality for pregnant women and children as well as interventions that have an impact on risk factors such as birth outcomes, wasting and stunting in children as well as risk factors among pregnant women such as anemia, micro-nutrient deficiencies and low body mass index. The model’s approach allows one to estimate the impact of scaling up coverage of a single or a set of interventions on maternal and child health as well as on the levels of the different risk factors. One can investigate the full set of interventions, causes of death and risk factors in LiST by looking at the LiST visualizer at www.livessavedtool.org.

The LiST database includes both national and subnational (where available) data since 1996, and is updated regularly. Development of the database and software were initially (2005–2013) guided by the Child Health Epidemiology Reference Group (CHERG) of the World Health Organization/UNICEF, which supported regular systematic reviews and updating of model assumptions and expansions of its content. Since 2013, the LiST team based at Johns Hopkins University has worked with various organizations to convene expert reviews and consultative meetings to ensure that LiST assumptions continue to reflect scientific advances [13]. The evidence supporting the assumptions used in LiST have been published at regular intervals in peer-reviewed journals [13].

In this paper we introduce a major LiST innovation: a web-based version of LiST. ‘LiST Online’ responds to issues identified in over 15 years of experience with the users of desktop version of the model. LiST Online increases access to the tool for users of desktops with limited memory, or running on computing platforms other than Windows [14], and does not require downloads of the full Spectrum software. LiST Online also has increased flexibility, allowing users to focus on specific time periods, interventions or outcomes of interest, or to develop bespoke applications, without losing the full computational power of the tool. LiST Online ensures that users have access to the most up-to-date release of the software, bringing the best available data to bear on their modeling questions. Finally, the layout, interface and visualization functions of LiST Online have been optimized for use on a range of devices (computers, tablets or phones), promoting frequent use to answer immediate questions. LiST Online has been subjected to three rounds of user testing to date, with positive results on ease of use – especially for new users.

LiST Online is organized into three inter-related tools:

1. EXPLORE DATA, which helps users visualize and critically review the input data they will use in developing their model;
2. MISSED OPPORTUNITIES, which assists users in assessing the relative impact of individual interventions or sets of interventions; and
3. PROJECTION, which provides flexible options for users to select default input data from the LiST database or to enter their own input data to estimate the lives saved through changes in intervention coverage.

We explain how each of these tools can be used to address core evaluation questions in the sections that follow.

## Using LiST to refine RMNCH&N program and evaluation plans

A first and fundamental core evaluation question is: ‘Does the program focus on interventions that will have the greatest impact in the program context?’. [Panel 1] Answering this question in the planning phase of an evaluation is important, because public health resources are scarce and many programs in the highest-mortality settings do not have health systems strong enough to implement complex strategies delivering multiple interventions at levels of quality sufficient to achieve impact. Although this may seem to be an obvious consideration, many programs are planned and implemented without a clear understanding of the impact they can expect from their program. Here we describe three specific ways in which using LiST to answer this question can strengthen programs and their evaluation.

First, modelled estimates of program impact can be used to refine the planned program. Program planners can use LiST, and especially the LiST Online EXPLORE DATA tool, to help them understand relevant parameters of their setting. LiST quickly and easily produces summaries of historic trends and current status for the major causes of death and relevant risk factors, as well as intervention coverage. The results provide the information needed to check on the extent to which planned program interventions target the major causes of death and can be expected to reduce mortality given current scientific evidence of efficacy. Program planners can then use the LiST Online MISSED OPPORTUNITIES tool to prioritize individual interventions based on their relative impact given local cause-of-death profiles, intervention efficacy and current coverage levels. Most RMNCH&N programs aim to increase coverage and quality for multiple interventions simultaneously. The LiST Online PROJECTION tool handles this complexity and allows users to build and explore scenarios containing alternative packages and approaches (e.g. a focus on preventive *vs*. curative interventions, or interventions delivered at facility *vs*. community level). LiST can also disaggregate the results to show how much of the estimated impact can be attributed to specific interventions or delivery approaches. This allows program planners to consider eliminating from the program those interventions that will have little or no impact, and introducing or prioritizing the interventions or approaches that yield the greatest impact – especially those that capitalize on potential economies of scale because they can be delivered through systems already being developed or strengthened by the program. For example, a program that seeks to improve the coverage and quality of an antenatal care package might find that almost all of their impact comes from one or two services, and that a tighter focus on these activities might reduce the costs and increase the impact of the overall program. LiST has been used in this way to improve the focus and expected impact of RMNCH&N programs in numerous countries [15] for the United Nations and other aid agencies [16–18].

Second, modelled estimates of program impact can be used to inform program expectations, set realistic program targets, and promote a shared understanding among implementers, evaluators and funders. Good practice in program planning includes setting quantitative targets, such as the number of mothers reached with birth care services, or the number of children vaccinated, as well as estimates of anticipated health impact. Unfortunately, most target-setting is based on little evidence, and given pressures to engage constituents or potential funders, the resulting targets are often unrealistically high. When these unrealistic targets are not achieved, the results can have negative effects on support and future funding for a program [19]. LiST modeling can help set the stage for realistic targets and expectations, providing an evidence-based rationale for a program plan. In addition to producing results on expected impact, program planners can use the LiST Online EXPLORE DATA tool to review both historical coverage trends in their setting and levels of coverage able to be achieved in other contexts with similar characteristics. This provides a useful starting point for setting realistic coverage targets and expectations. Using LiST to generate information for planning can promote a shared understanding among implementers, evaluators, and funders about what a program is aiming to do, the pathways through which these activities will result in population-level improvements in health and nutrition, and – as described in the next paragraph – the types of results that will be amenable to measurement and at what cost. Working proactively to build these shared expectations can help avoid vacillations in program funding and save lives.

Third, modelled estimates of program impact are needed to inform the development of an evaluation plan for the program. LiST results showing expected changes in coverage, health outcomes and risk factors can be used to define what needs to be measured in the evaluation, and whether it will be feasible (given sample size requirements) to capture expected changes within the time frame of the evaluation. For example, for a program aiming to increase exclusive breastfeeding rates among children under six months of age, LiST estimates that if the program reaches its target, the overall reduction in under-five mortality will be roughly five percent of the current under-five mortality rate of 60 per 1,000 live births or about 3 deaths per 1,000 live births. Based on this information, the evaluators can determine the size of the sample needed to measure the mortality reduction accurately, and work with program managers and evaluation funders to determine whether the investment needed to measure mortality change is justified. In many settings, obtaining accurate measurements of changes in intervention coverage and using modeling to estimate mortality impact is a more reasonable approach than conducting a population-based survey.

## Using list to estimate program impact and attribute effects

The final core evaluation question as proposed in this framework asks: ‘Is the expected impact of the program occurring? Why or why not?’ [Panel 1]. Responding to this question is the ultimate aim of a full effectiveness evaluation. In this section, we review how LiST can be used to assess and attribute the health and nutrition impact of a program in two different evaluation scenarios: as a *complement* to measured impact, and as a *replacement* for measured impact.

LiST can make important contributions even when an evaluation plan already includes high-quality measurements of the potential health and nutrition impact of a program. The estimates of lives saved produced by LiST from changes in measured coverage for specific interventions, using best-available estimates of the efficacy of those interventions for specific population subgroups and over time, including longer-term and indirect effects via risk factors. Estimates produced by LiST therefore reflect a best-case scenario of program impact, based on scientific evidence. Comparing what science predicts *should have happened* given measured changes in intervention coverage to what *did happen* as reflected in measured changes in mortality and nutritional status provides unprecedented learning opportunities for program planners and evaluators. For example, in settings or for subpopulations where large proportions of deaths can be addressed by interventions for which coverage can be measured accurately via household surveys (e.g. measles vaccination, oral rehydration therapy for diarrhea, first-line treatment for presumed malaria) [20], the match between LiST estimates and measured under-five mortality is often excellent. However, in settings where large proportions of deaths are due to causes for which accurate measurement of intervention coverage is difficult or impossible (e.g. maternal or neonatal deaths due to inadequate care around the time of birth) [21], there have been – in the past – larger gaps between modeled and measured estimates of program impact. This specific issue has largely been addressed by the inclusion of quality-adjusted coverage measurements in LiST, but illustrates how LiST has contributed to the growing focus on quality in RMNCH&N programs [22,23]. LiST has also proven useful in highlighting the importance of quality control in household surveys, the most common source of population-based measurements of both coverage and health and nutrition indicators [20,24]. For example, applications of LiST to estimate the impact of the national child survival program Tanzania showed wide discrepancies with the results of a national Demographic and Health Survey in 2010 [25,26]. These discrepancies disappeared when the results of LiST projections were compared to a later (2015–16) national DHS, confirming reports of quality control problems in the earlier survey [27].

Using LiST as a complement to measured results of impact modeling can produce estimates that spur important learning. This learning can include analyses of reasons for gap between the maximum program impact expected if the program is delivered according to best practices and the actual results, leading to identification and resolution of factors hindering implementation and evaluation design and measurement.

Perhaps the most important potential contribution of LiST to evaluations that also include measurement of impact is that LiST – unlike population-based surveys – can produce information about *how* program impact was achieved. Using measurements of coverage change, for multiple interventions, over time, and taking into account both demographic changes and changes in the susceptible population, LiST can estimate the relative contributions of individual or packaged interventions and the strategies used to deliver them to the target population for programs that produce measureable impact. LiST therefore provides a remarkable resource for those making decisions about how best to improve the health, survival and nutritional status of women and children.

For some programs, measuring impact is not feasible due to methodological, timing, financial or other constraints, and LiST provides a useful alternative. The effort and expense of measuring impact is rarely warranted if available data and documentation (e.g. coverage of key interventions) suggest that the program is unlikely to have had measureable impact on maternal or child health or nutrition within the period of the evaluation. When systematic efforts to answer evaluation questions in the framework addressing program focus, implementation and coverage (Panel 1) yield negative results, common sense suggests that scarce public health resources should not be used to measure impact, and evaluators should focus instead on understanding the reasons for limited progress earlier in the impact model. Here modelling of impact is not required but running the model based on the lack of change in coverage does provide evidence for making the decision to not try and collect impact data.

In many settings, accurate measurement of impact may not be feasible. Methodological constraints include difficulties in defining an appropriate comparison area or obtaining accurate measurements of outcomes. Timing constraints include situations in which impact results are needed immediately, and do not allow for sufficient periods for interventions to translate into changes in mortality or nutritional status. Examples include the longer-term benefits of implementing interventions that reduce stunting rates or interventions aimed at increasing the levels of vaccine coverage, both of which result in longer-term health benefits rarely able to be captured during a single survey designed to estimate impact. LiST can be used to estimate longer-term impacts of programming that extend beyond the period of the evaluation. Financial constraints include the high costs of conducting population-based surveys that include accurate measurement of mortality, with appropriate sample sizes, recall periods, and where necessary biometric technology. Accurate data on morbidity and mortality in geographically-defined program areas are rarely available from other sources, and the sample sizes needed to measure changes in these variables at population level are often prohibitively large. These challenges are not new, but precipitous drops in under-five and maternal mortality rates over the last 20 years [2,3] have made it increasingly difficult and expensive to measure change. Relative to health impact measures, the population-based estimates of intervention coverage needed to generate LiST estimates of impact are relatively inexpensive and feasible for most RMNCH&N projects to measure.

When funding for evaluations is limited and high-quality data on intervention coverage are available, LiST can produce defensible estimates of program impact at minimal cost. Panel 2 provides a general overview of how to use LiST to estimate program impact; the LiST Online PROJECTION tool provides easy access to the full computational power of LiST and allows adaptation for specific programs.

## Conclusions

Program evaluations are an important resource in efforts to save the lives of women and children. Well-designed program evaluations generate the evidence needed to improve programs and their effectiveness in saving lives, especially if guided by a new evaluation framework focused on five core evaluation questions (Panel 1). In this paper, we have explained why modeling should be a routine part of RMNCH&N program evaluations, and reviewed the historical underpinnings and continued development of the Lives Saved Tool (LiST) and its recent evolution into a web-based tool (LiST Online).

LiST is designed to capture best available evidence on the complex processes that affect RMNCH&N program effectiveness, and put it at the disposal of program planners, evaluators and funders. The latest iterations of the LiST model address three major impediments that have limited the effective use of modeling as a program evaluation tool in the past. First, LiST is designed to handle multiple causes of maternal, newborn and child deaths and the many potential interventions and external factors that act on these deaths, both directly or indirectly. Second, LiST captures the effects of a broad range of factors and interventions that determine changes in health outcomes and their synergistic and antagonistic interactions. Third, LiST – and particularly the new LiST Online – does not require technical expertise in modeling, and is therefore accessible to a broader range of users and can produce results quickly to inform decisions when they are being made. Finally, the new LiST Online has extended capabilities to support sub-national modeling and is designed to link with other programs and support the incorporation of the extensive LiST databases and powerful calculation engine into other modeling tools.

Those who want to familiarize themselves with LiST are encouraged to visit the LiST website at https://www.livessavedtool.org/. On-line training in LiST is available on the LiST website or via a series of in-person training workshops sponsored by the Government of Canada.

## Paper context

This paper focuses on roles modeling can play in evaluation of health programs in low- and middle-income countries. The paper discusses how modelling can be used to both guide evaluation data collection approaches by estimating expected impact and help estimate program impact in cases where there is little opportunity to measure impact due to financial or temporal constraints. The paper uses the LiST model as the exemplar of this type of work.

## References

[1] Hug L, Alexander M, You D, et al. National, regional, and global levels and trends in neonatal mortality between 1990 and 2017, with scenario-based projections to 2030: a systematic analysis. Lancet Glob Health. 2019;7:16–22. 10.1016/S2214-109X(19)30163-9 PMC6527519 31097275

[2] UN Inter-agency Group for Child Mortality Estimation (IGME. Levels & Trends in Child Mortality. New York: IGME Report 2019. [cited 2020 Sept 1]. Available from: https://www.unicef.org/media/60561/file/UN-IGME-child-mortality-report-2019.pdf

[3] World Health Organization Trends in maternal mortality 2000 to 2017: estimates Geneva: WHO, UNICEF, UNFPA, World Bank Group and the United Nations Population Division. 2019 [cited 2020 Sept 1]. Available from: file:///C:/Users/JBryce/Downloads/9789241516488-eng.pdf

[4] Raifman JG, Lam F, Keller JM, et al. Evaluating evaluations: assessing the quality of Aid Agency evaluations in Global Health. CGD Working Paper 461. Washington, DC: Center for Global Development. 2017 [cited 2020 Apr 28]. Available from: https://www.cgdev.org/publication/evaluating-evaluations-assessing-quality-aid-agency-evaluations-global-health

[5] Amouzou A, Bryce J, Walker N; the JHU RADAR Team. Strengthening effectiveness evaluations to improve programs for women, children, and adolescents. Paper 1 in this Supplement. 10.1080/16549716.2021.2006423 PMC9481099 36098952

[6] Garnett GP, Cousens S, Hallett TB, et al. Mathematical models in the evaluation of health programmes. Lancet. 2011;378:515–525. 21481448 10.1016/S0140-6736(10)61505-X

[7] Jones G, Steele R, Bhatta Z, et al.; the Bellagio Child Survival Study Group. How many child deaths can we prevent this year? Lancet. 2003;362:65–71. 12853204 10.1016/S0140-6736(03)13811-1

[8] Avenir Health. Spectrum. [cited 2020 Jun 28]. Available from: https://www.avenirhealth.org/software-spectrum.php

[9] Lancet Series on Maternal and Child Nutrition. [cited 2020 Jun 28]. Available from: https://www.thelancet.com/series/maternal-and-child-nutrition

[10] Lancet series on Maternal and Child Undernutrition. 2018 [cited 2020 Jun 28]. Available from: https://www.thelancet.com/series/maternal-and-child-undernutrition

[11] Kanyangarara M, Chou VB, Creanga AA, et al. Linking household and health facility surveys to assess obstetric service availability, readiness and coverage: evidence from 17 low- and middle-income countries. J Glob Health. 2018;8. DOI:10.7189/jogh.08.010603 PMC5963736 29862026

[12] Kanyangarara M, Walker N, Boerma T. Gaps in the implementation of antenatal syphilis detection and treatment in health facilities across sub-Saharan Africa. PLoS ONE. 2018;13:e0198622. 29856849 10.1371/journal.pone.0198622 PMC5983468

[13] Research: Scientific Basis of List. [cited 2020 Sept 2]. Available from: https://www.livessavedtool.org/research

[14] Encyclopaedia Britannia. Microsoft Windows. [updated 28 May 2020; cited 2020 Jun 30]. Available from: https://www.britannica.com/technology/Windows-OS

[15] Bryce J, Friberg IK, Kraushaar D, et al. LiST as a catalyst in program planning: experiences from Burkina Faso, Ghana and Malawi. Int J Epidemiol. 2010;39:i40–i47. 20348125 10.1093/ije/dyq020 PMC2845859

[16] Save the Children K. Ending child deaths from Pneumonia and Diarrhoea: one is too many. New York: UNICEF; 2016.

[17] Fighting for breath: a call to action on childhood pneumonia. Save the Children UK, London. 2017.

[18] US Agency for International Development. Acting on the Call - Ending preventable child and maternal deaths: a focus on equity. Washington (DC): USAID; 2016 June.

[19] Pritchett L. It pays to be ignorant: a simple political economy of rigorous program evaluation. J Policy Reform. 2002;5:251–269.

[20] Munos MK, Stanton CK, Bryce J. Core group for improving coverage measurement for MNCH. Improving coverage measurement for reproductive, maternal, neonatal and child health: gaps and opportunities. J Glob Health. 2017;7:010801. 28607675 10.7189/jogh.07.010801 PMC5460400

[21] Munos M, Maïga A, Sawadogo-Lewis T, et al. The RADAR coverage tool: developing a toolkit for rigorous household surveys for reproductive, maternal, newborn, and child health & nutrition indicators. Paper in the supplement 10.1080/16549716.2021.2006419 PMC9481084 36098955

[22] Goyet S, Broch-Alvarez V, Becker C. Quality improvement in maternal and newborn healthcare: lessons from programmes supported by the German development organisation in Africa and Asia. BMJ Glob Health. 2019;4:e001562. 10.1136/bmjgh-2019-001562 PMC6747907 31565404

[23] Marx M, Frost E, Hazel E, et al. Tools and methods to measure the quality of care for maternal, neonatal, child, reproductive health and nutrition programs: guidance from use in four sub Saharan African Countries through the RADAR project. Paper in supplement.

[24] Do M, Micah A, Brondi L, et al. Linking household and facility data for better coverage measures in reproductive, maternal, newborn, and child health care: systematic review. J Glob Health. 2016;6:020501. 27606060 10.7189/jogh.06.020501 PMC5012234

[25] Afnan-Holmes H, Magoma M, John T, et al. Tanzania’s Countdown to 2015: an analysis of two decades of progress and gaps for reproductive, maternal, newborn, and child health, to inform priorities for post-2015. Lancet Glob Health. 2015;3:e396–e409. 26087986 10.1016/S2214-109X(15)00059-5

[26] National Bureau of Statistics - NBS/Tanzania and ICF Macro. Tanzania Demographic and Health Survey 2010. Dar es Salaam Tanzania: NBS/Tanzania and ICF Macro; 2011.

[27] MoHCDGEC/Tanzania Mainland, Ministry of Health - MoH/Zanzibar, National Bureau of Statistics - NBS/Tanzania, Office of Chief Government Statistician - OCGS/Zanzibar, and ICF. Tanzania Demographic and Health Survey and Malaria Indicator Survey (TDHS-MIS) 2015-16. Dar es Salaam/Tanzania: MoHCDGEC, MoH, NBS, OCGS, and ICF; 2016.

